# CuBr_2_ complexes with 3,5-disubstituted pyridine ligands

**DOI:** 10.1107/S2056989025001343

**Published:** 2025-02-18

**Authors:** Christopher P. Landee, Diane A. Dickie, Mark M. Turnbull

**Affiliations:** a Dept. of Physics, Clark University, 950 Main St., Worcester, MA 01610, USA; b Dept. of Chemistry, University of Virginia, McCormack Rdl., Charlottesville, VA 22904, USA; c Carlson School of Chemistry and Biochemistry, Clark University, 950 Main St., Worcester, MA 01610, USA; Vienna University of Technology, Austria

**Keywords:** crystal structure, copper(II) bromide, 3,5-di­chloro­pyridine, 3,5-di­methyl­pyridine

## Abstract

Reaction of copper(II) bromide with 3,5-di­chloro­pyridine (3,5-Cl_2_py) or 3,5-di­methyl­pyridine (3,5-Me_2_py) led to the isolation of the coordination polymers *catena*-poly[[bis­(3,5-di­chloro­pyridine)­copper(II)]-di-μ-bromido], [CuBr_2_(C_5_H_3_Cl_2_N)_2_]_*n*_ or [CuBr_2_(3,5-Cl_2_py)_2_]_*n*_ (**1**), and *catena*-poly[[bis­(3,5-di­methyl­pyridine)­copper(II)]-di-μ-bromido], [CuBr_2_(C_7_H_9_N)_2_]_*n*_ or [CuBr_2_(3,5-Me_2_py)_2_]_*n*_ (**2**), respectively. The structures are characterized by bibromide-bridged chains [*d*(av.)_Cu⋯Cu_ = 3.93 (9) Å]. In **1**, the chains are linked perpendicular to the *a* axis by non-classical hydrogen bonds and halogen bonds, while in **2**, only non-classical hydrogen bonds are observed.

## Abstract 2

Compounds **1** and **2** are similar coordination polymers of bibromide bridged chains of Cu^II^ ions with 3,5-disubstituted pyridine mol­ecules in the axial sites. The chains lie parallel to the *a* axis and are linked into a tri-periodic network *via* non-classical hydrogen bonds.

## 1. Chemical context

The introduction of random features in a structure may have significant and unique effects on the physical properties of materials. As such, attempts have been made to introduce randomness into the structures of solids by chemists and physicists through modification of the crystal structure (Anderson, 1958 ▸; Mackenzie, 1964 ▸) with particular inter­est in its applications for quantum information (Khrennikov, 2016 ▸; Feng *et al.*, 2025 ▸) and band theory (Coey *et al.*, 2005 ▸; Murugesan *et al.*, 2025 ▸). Specific to the field of magnetism, the effects of randomness on valence-bond solids (Kimchi *et al.*, 2018 ▸) and spin glasses (Toulouse, 1986 ▸) have been areas of focus.
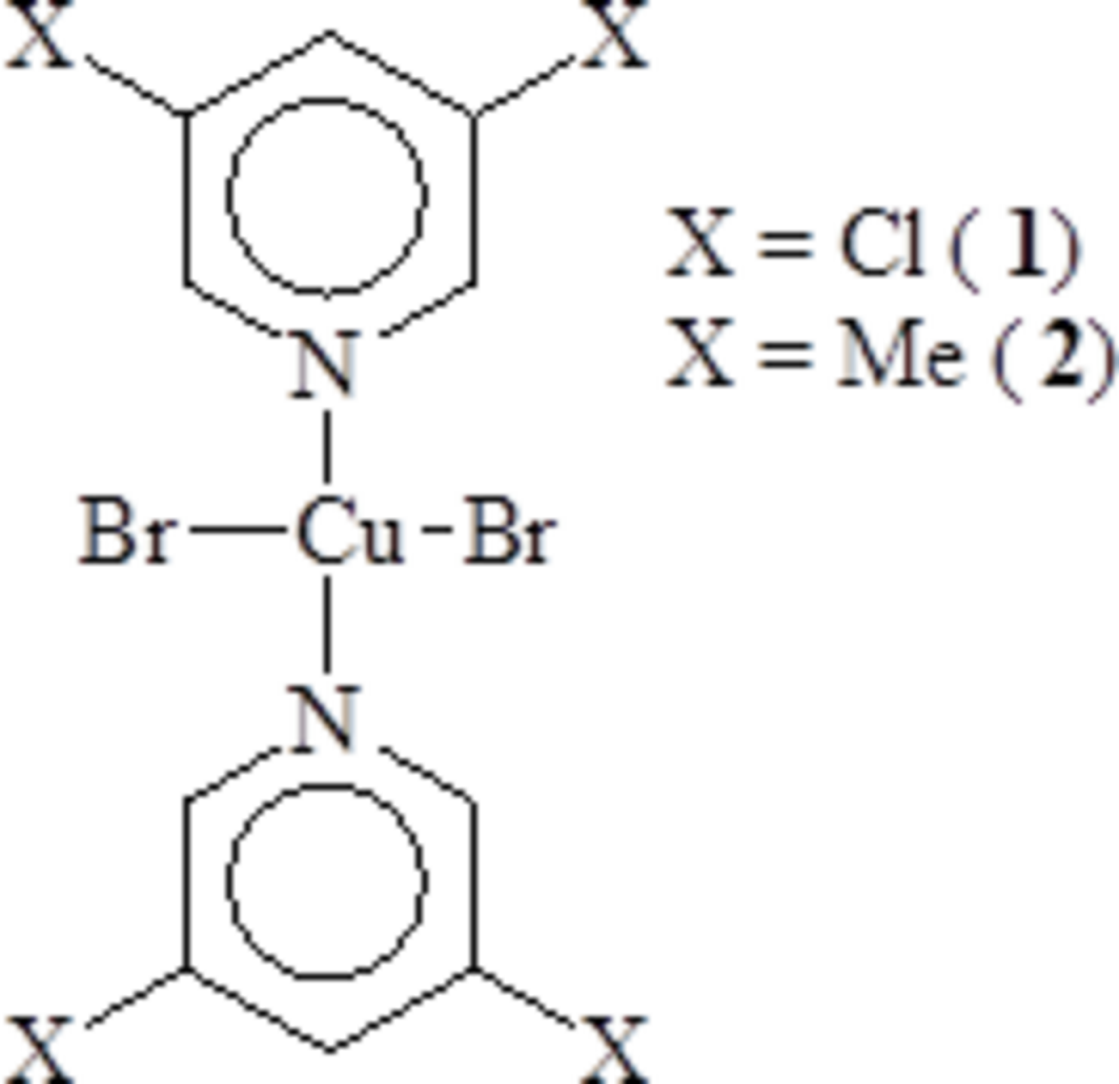


We have been studying the mechanism of magnetic superexchange for some time through the production of families of complexes of transition metal ions, in particular those containing substituted pyridine species as ligands (Graci *et al.*, 2024*a* ▸,*b* ▸; Monroe *et al.*, 2024 ▸; Atkinson *et al.*, 2024 ▸) or charge-balancing cations (Graci *et al.*, 2024*a* ▸; Bellesis *et al.*, 2024 ▸). In the hope of introducing randomness into such compounds, we have examined systems where we hoped that structurally similar compounds with subtly different ligands would allow the production of solid solutions of low-dimensional coordin­ation polymers. One such possible paring included Cu^II^ bibromide-bridged chains with ancillary pyridine ligands of different, but similar, substitution. Here we report the preparation and structures of the CuBr_2_*L*_2_ complexes with *L* = 3,5-di­chloro­pyridine (**1**) or 3,5-di­methyl­pyridine (**2**).

## 2. Structural commentary

**Figure 1:**
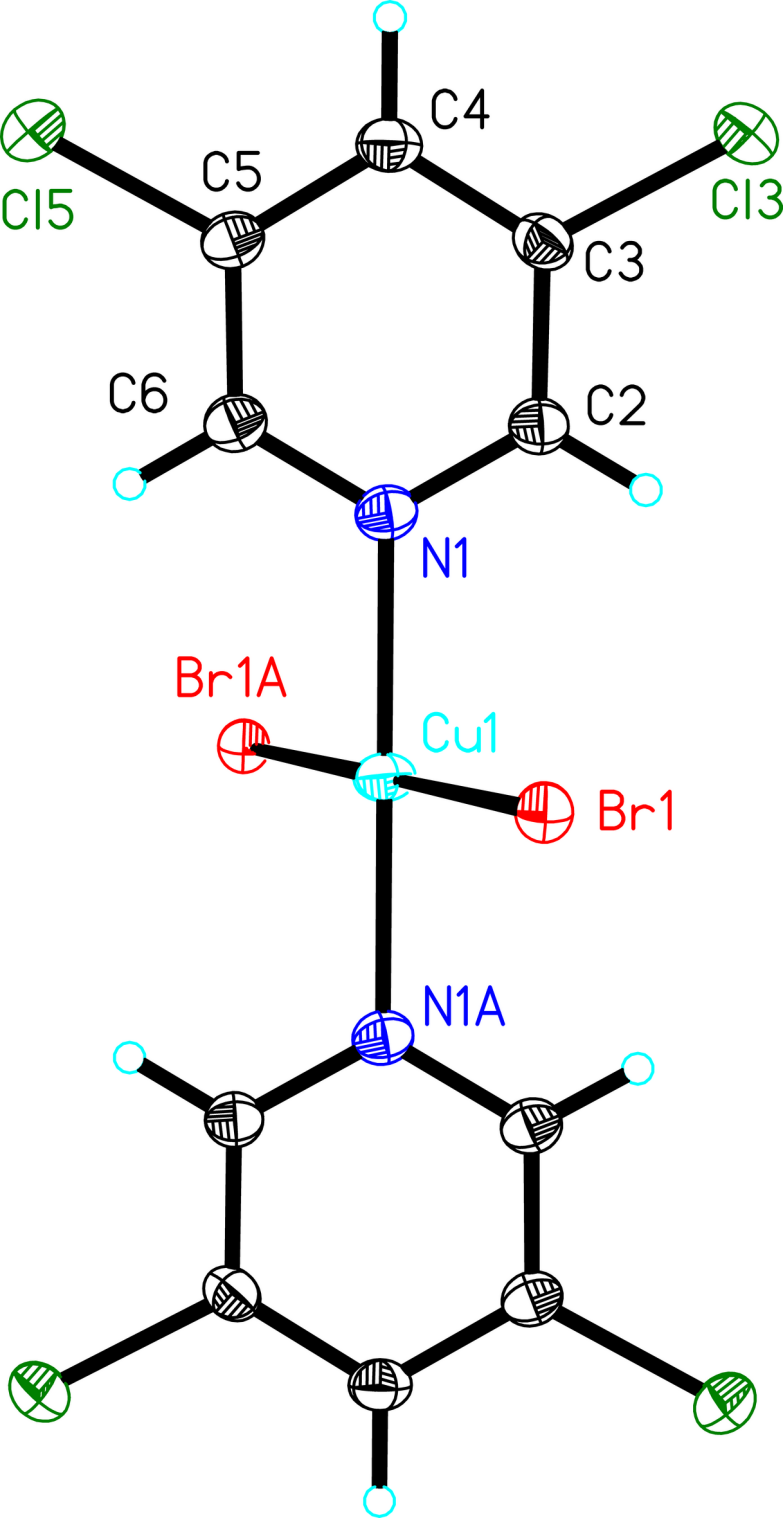
The mol­ecular unit of **1** showing displacement ellipsoids at the 50% probability level (hydrogen atoms are shown as spheres of arbitrary size). Only the asymmetric unit and Cu coordination sphere are labeled. [Symmetry code: (A) 1 − *x*, 1 − *y*, 2 − *z*.]

The asymmetric unit of [CuBr_2_(3,5-Cl_2_py)_2_]_*n*_ (**1**) [3,5-Cl_2_py = 3,5-di­chloro­pyridine) is composed of one 3,5-Cl_2_py mol­ecule, one bromide ion and one Cu^II^ ion, which is located on an inversion center rendering all *trans*-bonds 180° as required by symmetry. The mol­ecular unit is shown in Fig. 1 ▸. Selected bond lengths and angles are provided in Table 1 ▸. The coordination environment around the Cu^II^ ion is nearly square-planar [∠_Br1—Cu1—N1_ = 89.66 (10)°]. The copper coordination sphere is planar, also as required by symmetry, and the plane of the pyridine ring (mean deviation of constituent atoms = 0.0086 Å) is inclined by 58.1 (1)° relative to that plane. The chlorine atoms are displaced slightly (∼0.05 Å) to opposite faces of the pyridine ring.

**Figure 2:**
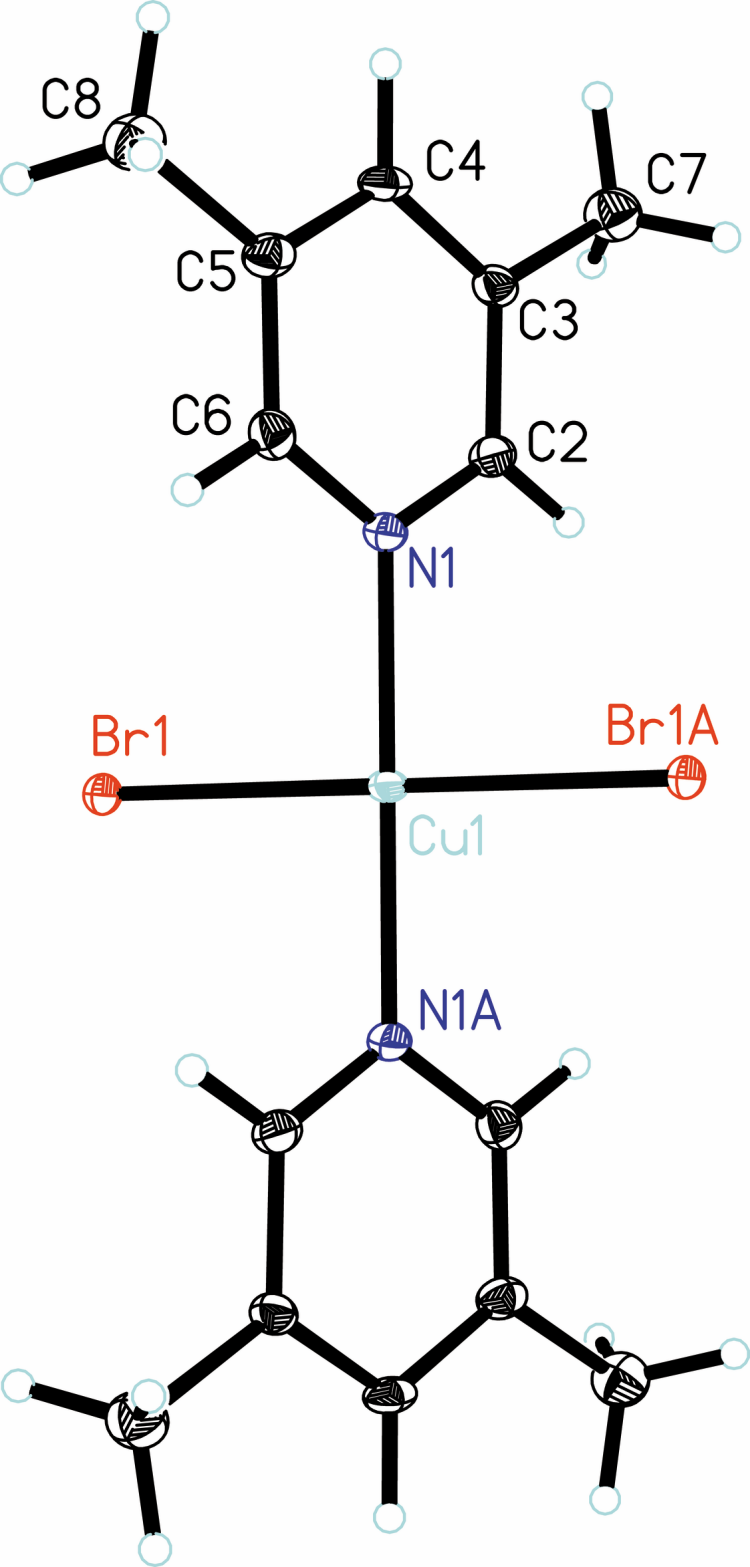
The mol­ecular unit of **2** showing displacement ellipsoids at the 50% probability level (hydrogen atoms are shown as spheres of arbitrary size). Only the asymmetric unit and Cu coordination sphere are labeled. [Symmetry code: (A) 1 − *x*, 1 − *y*, 2 − *z*.]

[CuBr_2_(3,5-Me_2_py)_2_]_*n*_ (**2**) [3,5-Me_2_py = 3,5-dimthyl­pyridine) is structurally very similar to **1**, with one 3,5-Me_2_py mol­ecule, one bromide ion and one Cu^II^ ion comprising the asymmetric unit (Fig. 2 ▸). Selected bond lengths and angles are provided in Table 2 ▸. The coordination environment around the Cu^II^ ion is again nearly square-planar [∠_Br1—Cu1—N1_ = 90.27 (12)°]with the plane of the pyridine ring (mean deviation of the constituent atoms = 0.0095 Å) inclined by 60.8 (1)° relative to the Cu coordination plane. The carbon atoms of the methyl groups are displaced slightly to opposite faces of the plane of the pyridine ring (C7, ∼0.02 Å; C8, ∼0.01 Å).

## 3. Supra­molecular features

**Figure 3:**
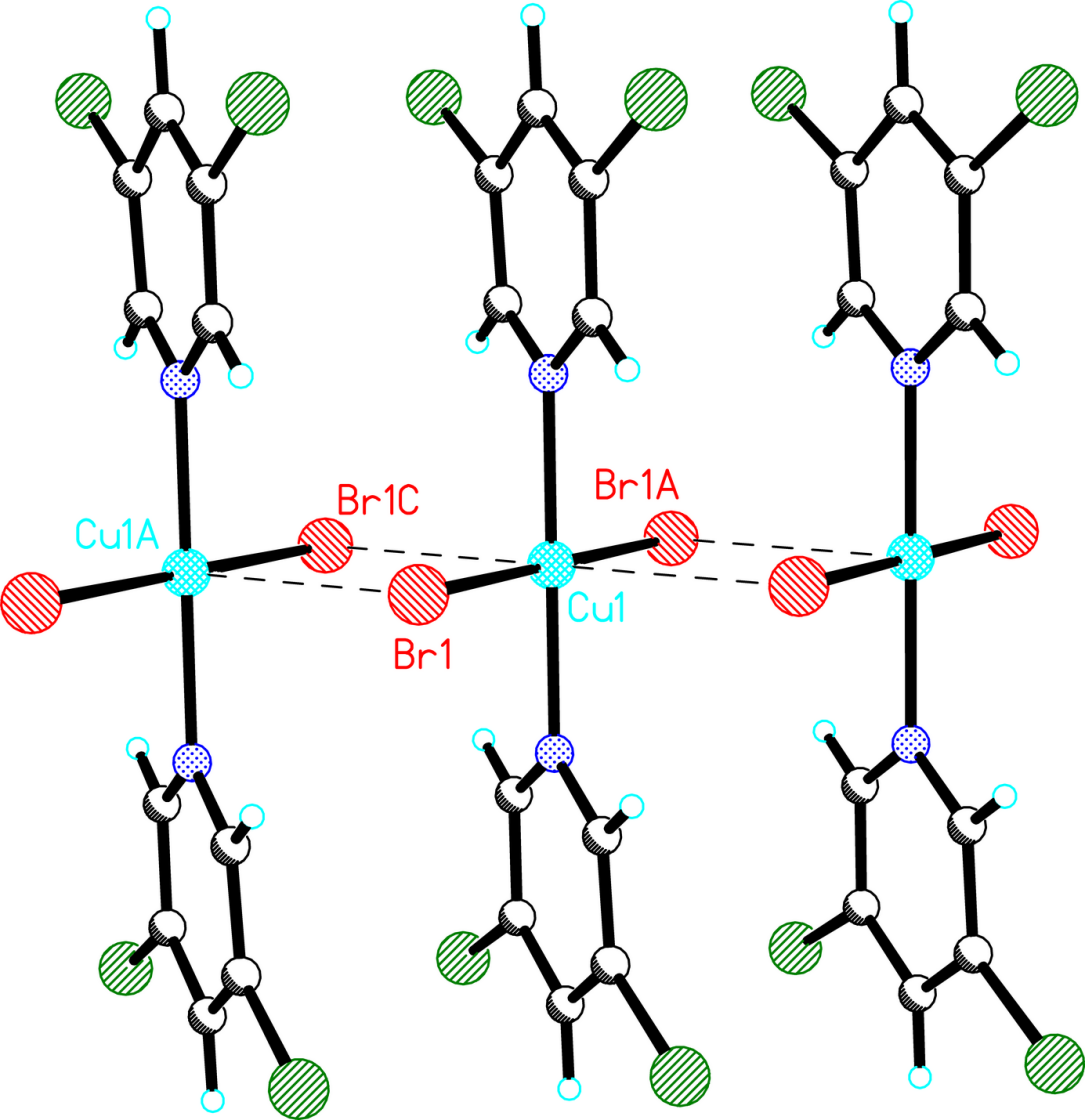
The chain structure of **1** viewed parallel to the *bc* face diagonal (*a* axis horizontal). [Symmetry codes: (A) *x* − 1, *y*, *z*; (C) −*x*, 1 − *y*, 2 − *z*.]

**Figure 4:**
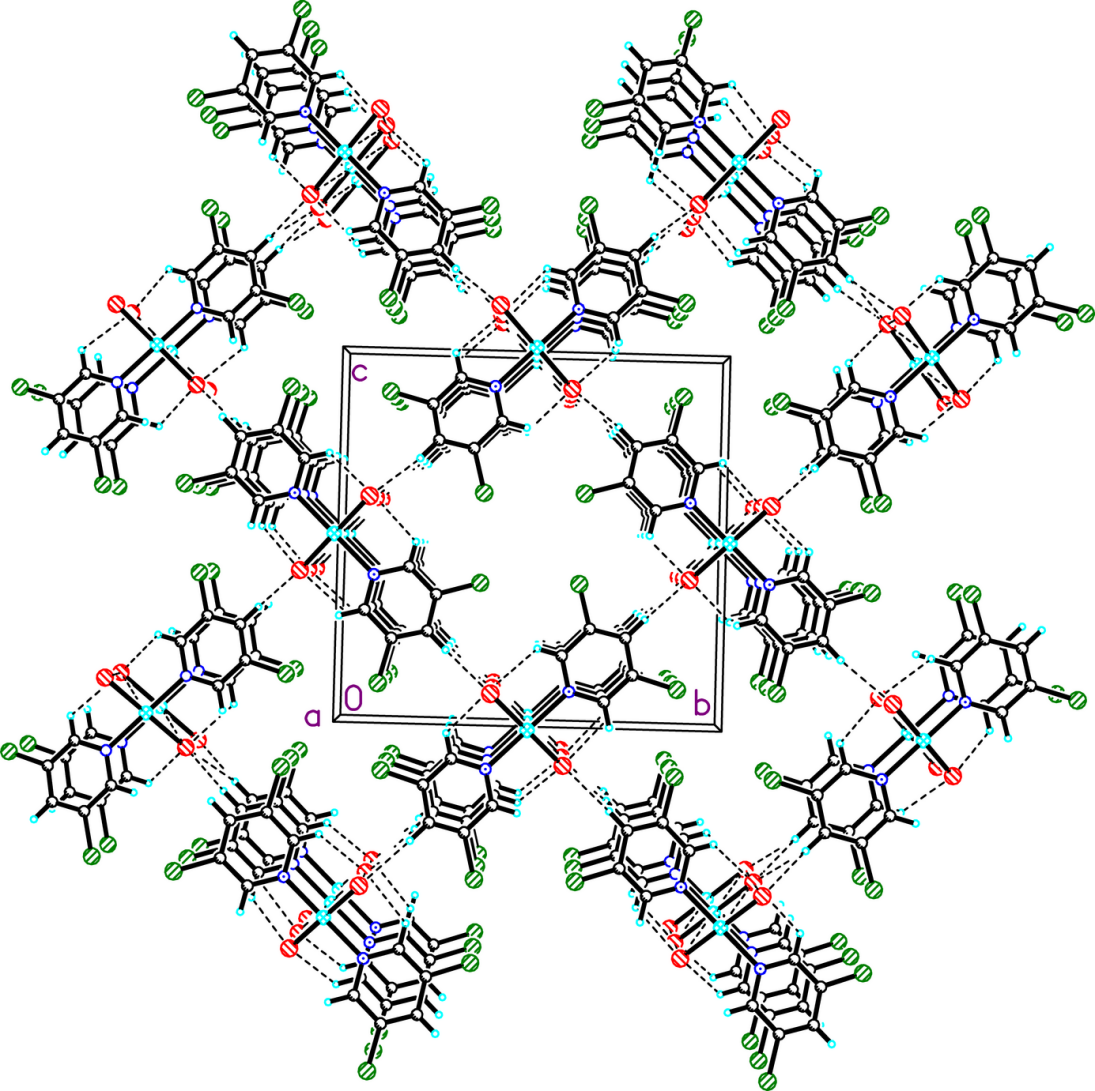
The crystal structure of **1** viewed parallel to the *a* axis (chain axis). Dashed lines represent hydrogen bonds.

Mol­ecules of **1** are linked into chains parallel to the *a* axis via non-symmetrically bridging bromide ions (Fig. 3 ▸). Each bromide ion exhibits a long Br1⋯Cu1*A* contact of 3.031 (5) Å with a corresponding Cu1—Br1⋯Cu1^A^ angle of 89.6 (2)° and a Br1—Cu1⋯Br1^C^ angle of 90.4 (2) (supplementary as required by symmetry; symmetry codes refer to Fig. 3 ▸). The chains are further stabilized by weak, non-classical hydrogen bonds between the hydrogen atoms *ortho* to the pyridine nitro­gen atoms and bromide ions of adjacent mol­ecules in the chain (Table 3 ▸). Inter­chain inter­actions occur *via* non-classical hydrogen bonds (Table 3 ▸, Fig. 4 ▸) between the C4—H4 group and a bromide ion of a neighboring chain related by a 2_1_-screw axis. Additional inter­chain stabilization is provided by Type II halogen bonds between chlorine atoms of adjacent chains [*d*_Cl3⋯Cl5A_ = 3.601 (3) Å, ∠_C3—Cl3⋯Cl5B_ = 104.6 (2)°, ∠_Cl3⋯Cl5B—C5B_ = 151.0 (2)°].

**Figure 5:**
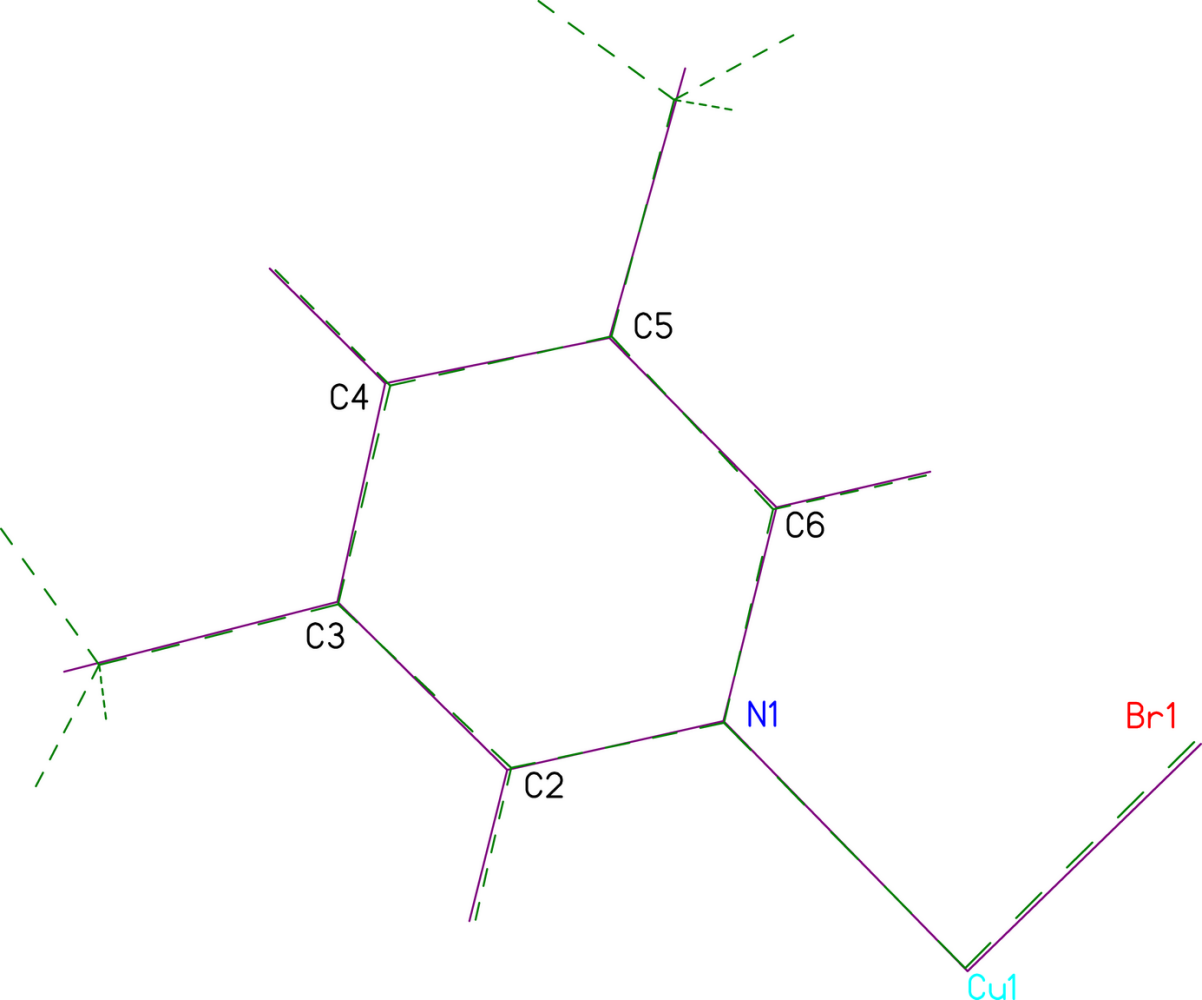
An overlay of the asymmetric units of **1** (solid bonds) and **2** (dashed bonds). The overlay was created using the best fit of the species Cu1, Br1 and N1 from the two structures.

Compound **2** is structurally similar to **1**. The semi-coordinate bridging bromide ions make contacts of 3.213 (5) Å between unit-cell translated mol­ecules, again parallel to the *a* axis. These are significantly longer (∼0.2 Å) than observed in **1** as a result of the larger methyl substituents. The corresponding angles are ∠_Cu1—Br1⋯Cu1A_ = 88.8 (2)° and ∠_Br1—Cu1⋯Br1C_ = 91.2 (2)°, showing a larger deviation from 90° compared with **1**. Further intra­chain stabilization is again provided *via* non-classical hydrogen bonds (Table 4 ▸). Unsurprisingly, again the bulk of the methyl groups forces increased separations and an equivalent non-classical hydrogen bond between C4—H4 and a neighboring chain bromide ion becomes significantly weaker [*d* = 3.742 (5) Å] compared to **1**, although the overall inter­chain geometry is maintained. The absence of the chlorine atoms, and hence the halogen bonds, is likely also partially responsible for the increased separation between chains. The structurally similar nature of the asymmetric units is clearly seen in the overlay of the two structures (Fig. 5 ▸).

The structure of **2** has previously been reported based on film data (Ooijen *et al.*, 1979 ▸) with a reliability factor *R* = 13.0 at 295 K. In the prior study, the positions of hydrogen atoms were not included in the final refinement. The Cu—Br and Cu—N bond lengths and Cu⋯Br contact distances are all somewhat longer (3.286 Å) as would be expected at the higher temperature. The reported Br—Cu—N angle deviates slightly more from 90° (∼0.6 °) while the Cu—Br⋯Cu bridging angle is significantly closer to 90° (89.96°) compared to the refined crystal structure model of **2**.

Regretably, attempts to prepare crystals with mixed 3,5-di­chloro­pyridine and 3,5-di­methyl­pyridine were unsuccessful in spite of the structurally similar nature of the individual complexes.

## 4. Database survey

A significant number of complexes of the general formula Cu*X*_2_(s-py)_2_ has been reported (where s-py represents a substituted pyridine ligand) based upon a survey of the Cambridge Structure Database (CSD, version 5.46, update November 2024; Groom *et al.*, 2016 ▸). With s = H, both the chloride and bromide complexes are known (Morosin, 1975 ▸). Those compounds and compounds with substituents in the 4-position tend to form bi-bridged chains similar to **1** and **2** with substituents including alkyl groups (Laing & Carr, 1971 ▸; Marsh *et al.*, 1981 ▸; Matshwele *et al.*, 2022 ▸), alk­oxy groups (Gungor, 2021 ▸), halogens (Vitorica-Yrezabel *et al.*, 2011 ▸) and carboxyl­ate derivatives (Fellows & Prior, 2017 ▸; Ahadi *et al.*, 2015 ▸; Zhang *et al.*, 1997 ▸; Hearne *et al.*, 2019 ▸; Heine *et al.*, 2020*a* ▸; Ma *et al.*, 2010 ▸). The same is generally true for substituents in the 3/5 positions (with or without a substituent also in the 4-position), with substituents such as hy­droxy (Segedin *et al.*, 2008 ▸), amino (Lah & Leban, 2005 ▸), alkyl (Bondarenko *et al.*, 2021 ▸; Awwadi, 2013 ▸), aryl (Richardson *et al.*, 2018 ▸), halogens (Awwadi *et al.*, 2006 ▸, 2011 ▸; Mínguez Espallargas *et al.*, 2006 ▸; Puttreddy *et al.*, 2018 ▸) and carboxyl­ate derivatives (Fellows & Prior, 2017 ▸; Chen *et al.*, 2011 ▸). However, this can be disrupted by the presence of substituents that can coordinate to the open coordination sites at the Cu^II^ ion (Li *et al.*, 2004 ▸; Zhang *et al.*, 2004 ▸), which may result in polymorphs (Heine *et al.*, 2020*b* ▸). Bulky substituents in the 2-position may result in the formation of dimers (Forman *et al.*, 2015 ▸; Huynh *et al.*, 2023 ▸; Herringer *et al.*, 2011 ▸) rather than extended chains, or simply isolated complexes (Lennartson *et al.*, 2007 ▸; Vural & İdil, 2019 ▸; Aguirrechu-Comerón *et al.*, 2015 ▸). The effects of multiple substituents has been recently described (Dubois *et al.*, 2018 ▸, 2019 ▸).

## 5. Synthesis and crystallization

Compound **1**: CuBr_2_ (0.221 g, 0.99 mmol) and 3,5-di­chloro­pyridine (0.298 g, 2.01 mmol) were dissolved in 25 ml of aceto­nitrile, covered with parafilm with a few holes introduced and left to crystallize at room temperature. After ∼3 weeks, green needles were isolated by filtration, washed quickly with cold aceto­nitrile and allowed to air-dry to give 0.189 g (37%).

Compound **2**: CuBr_2_ (0.225 g, 1.01 mmol) and 3,5-di­methyl­pyridine (0.221 g, 2.06 mmol) were dissolved in 20 ml of aceto­nitrile with gentle warming, covered with parafilm with a few holes introduced and left to crystallize at room temperature. After ∼3 weeks, green needles were isolated by filtration, washed quickly with cold aceto­nitrile and allowed to air-dry to give 0.146 g (33%).

## 6. Refinement

Crystal data, data collection and structure refinement details are summarized in Table 5 ▸. Hydrogen atoms bonded to carbon atoms were placed geometrically and refined with a riding model with *U*_iso_(H) = 1.2(C). The crystal of **2** under investigation was a four-component twin with refined fractional volume contributions of 0.5063, 0.4350, 0.0381 and 0.0206.

## Supplementary Material

Crystal structure: contains datablock(s) 1, 2. DOI: 10.1107/S2056989025001343/wm5752sup1.cif

Structure factors: contains datablock(s) 1. DOI: 10.1107/S2056989025001343/wm57521sup2.hkl

Structure factors: contains datablock(s) 2. DOI: 10.1107/S2056989025001343/wm57522sup3.hkl

CCDC references: 2423676, 2423675

Additional supporting information:  crystallographic information; 3D view; checkCIF report

## Figures and Tables

**Table 1:** Selected geometric parameters (Å, °) for **1**[Chem scheme1]

Cu1—N1	2.039 (3)	Cu1—Br1	2.4246 (4)
			
N1—Cu1—Br1	89.66 (10)		

**Table 2:** Selected geometric parameters (Å, °) for **2**[Chem scheme1]

Cu1—N1	2.007 (4)	Cu1—Br1	2.4350 (5)
			
N1—Cu1—Br1	90.27 (12)		

**Table 3:** Hydrogen-bond geometry (Å, °) for **1**[Chem scheme1]

*D*—H⋯*A*	*D*—H	H⋯*A*	*D*⋯*A*	*D*—H⋯*A*
C2—H2⋯Br1^i^	0.95	2.83	3.472 (4)	126
C4—H4⋯Br1^ii^	0.95	2.86	3.622 (4)	138
C6—H6⋯Br1^iii^	0.95	2.82	3.441 (4)	124

Symmetry codes: (i) 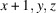; (ii) 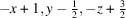; (iii) 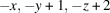.

**Table 4:** Hydrogen-bond geometry (Å, °) for **2**[Chem scheme1]

*D*—H⋯*A*	*D*—H	H⋯*A*	*D*⋯*A*	*D*—H⋯*A*
C2—H2⋯Br1^i^	0.95	3.12	3.406 (5)	100
C6—H6⋯Br1^ii^	0.95	2.83	3.515 (5)	130

Symmetry codes: (i) 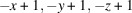; (ii) 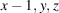.

**Table 5:** Experimental details

	**1**	**2**
Crystal data		
Chemical formula	[CuBr_2_(C_5_H_3_Cl_2_N)_2_]	[CuBr_2_(C_7_H_9_N)_2_]
*M*_r_	519.33	437.66
Crystal system, space group	Monoclinic, *P*2_1_/*c*	Monoclinic, *P*2_1_/*c*
Temperature (K)	120	100
*a*, *b*, *c* (Å)	3.86683 (17), 14.1943 (6), 13.7347 (6)	3.9901 (2), 14.2902 (9), 13.8338 (8)
β (°)	91.453 (4)	93.638 (2)
*V* (Å^3^)	753.62 (5)	787.20 (8)
*Z*	2	2
Radiation type	Cu *K*α	Mo *K*α
μ (mm^−1^)	14.67	6.45
Crystal size (mm)	0.20 × 0.04 × 0.04	0.46 × 0.03 × 0.03

Data collection		
Diffractometer	SuperNova, Dual, Cu at zero, Atlas	Bruker APEXII CCD
Absorption correction	Multi-scan (*CrysAlis PRO*; Agilent Technologies, 2011 ▸	Multi-scan (*TWINABS*; Sheldrick, 2012 ▸)
*T*_min_, *T*_max_	0.531, 1.000	0.650, 0.746
No. of measured, independent and observed [*I* > 2σ(*I*)] reflections	8288, 1513, 1406	3775, 3775, 3425
*R*_int_	0.052	0.058
(sin θ/λ)_max_ (Å^−1^)	0.623	0.667

Refinement		
*R*[*F*^2^ > 2σ(*F*^2^)], *wR*(*F*^2^), *S*	0.039, 0.102, 1.05	0.044, 0.087, 1.05
No. of reflections	1513	3775
No. of parameters	88	91
H-atom treatment	H-atom parameters constrained	H-atom parameters constrained
Δρ_max_, Δρ_min_ (e Å^−3^)	1.15, −0.85	0.69, −0.64

Computer programs: *CrysAlis PRO* (Agilent Technologies, 2011 ▸), *APEX4* and *SAINT* (Bruker, 2022 ▸), *SHELXS* (Sheldrick, 2008 ▸), *SHELXL* (Sheldrick, 2015 ▸), *SHELXTL* (Sheldrick, 2008 ▸) and *publCIF* (Westrip, 2010 ▸).

## supplementary crystallographic information

### catena-Poly[[bis(3,5-dichloropyridine)copper(II)]-di-µ-bromido] (1) Crystal data

**Table:**

[CuBr_2_(C_5_H_3_Cl_2_N)_2_]	*F*(000) = 494
*M**_r_* = 519.33	*D*_x_ = 2.289 Mg m^−^^3^
Monoclinic, *P*2_1_/*c*	Cu *K*α radiation, λ = 1.54184 Å
*a* = 3.86683 (17) Å	Cell parameters from 4275 reflections
*b* = 14.1943 (6) Å	θ = 3.1–73.6°
*c* = 13.7347 (6) Å	µ = 14.67 mm^−^^1^
β = 91.453 (4)°	*T* = 120 K
*V* = 753.62 (5) Å^3^	Rod, green
*Z* = 2	0.20 × 0.04 × 0.04 mm

### catena-Poly[[bis(3,5-dichloropyridine)copper(II)]-di-µ-bromido] (1) Data collection

**Table:**

SuperNova, Dual, Cu at zero, Atlas diffractometer	1513 independent reflections
Radiation source: SuperNova (Cu) X-ray Source	1406 reflections with *I* > 2σ(*I*)
Mirror monochromator	*R*_int_ = 0.052
Detector resolution: 10.6501 pixels mm^-1^	θ_max_ = 73.8°, θ_min_ = 4.5°
ω scans	*h* = −4→4
Absorption correction: multi-scan (CrysAlisPro; Agilent Technologies, 2011	*k* = −17→17
*T*_min_ = 0.531, *T*_max_ = 1.000	*l* = −16→17
8288 measured reflections	

### catena-Poly[[bis(3,5-dichloropyridine)copper(II)]-di-µ-bromido] (1) Refinement

**Table:**

Refinement on *F*^2^	Primary atom site location: structure-invariant direct methods
Least-squares matrix: full	Secondary atom site location: difference Fourier map
*R*[*F*^2^ > 2σ(*F*^2^)] = 0.039	Hydrogen site location: inferred from neighbouring sites
*wR*(*F*^2^) = 0.102	H-atom parameters constrained
*S* = 1.05	*w* = 1/[σ^2^(*F*_o_^2^) + (0.0663*P*)^2^ + 1.7392*P*] where *P* = (*F*_o_^2^ + 2*F*_c_^2^)/3
1513 reflections	(Δ/σ)_max_ = 0.001
88 parameters	Δρ_max_ = 1.15 e Å^−^^3^
0 restraints	Δρ_min_ = −0.85 e Å^−^^3^

### catena-Poly[[bis(3,5-dichloropyridine)copper(II)]-di-µ-bromido] (1) Special details

**Table:**

Geometry. All esds (except the esd in the dihedral angle between two l.s. planes) are estimated using the full covariance matrix. The cell esds are taken into account individually in the estimation of esds in distances, angles and torsion angles; correlations between esds in cell parameters are only used when they are defined by crystal symmetry. An approximate (isotropic) treatment of cell esds is used for estimating esds involving l.s. planes.

### catena-Poly[[bis(3,5-dichloropyridine)copper(II)]-di-µ-bromido] (1) Fractional atomic coordinates and isotropic or equivalent isotropic displacement parameters (Å^2^)

**Table:**

	*x*	*y*	*z*	*U*_iso_*/*U*_eq_	
Cu1	0.500000	0.500000	1.000000	0.0193 (2)	
Br1	0.10173 (9)	0.59337 (3)	0.90079 (3)	0.01846 (17)	
N1	0.4871 (9)	0.3973 (2)	0.8962 (3)	0.0201 (7)	
C2	0.5726 (10)	0.4173 (3)	0.8042 (3)	0.0200 (8)	
H2	0.642863	0.479406	0.788200	0.024*	
C3	0.5596 (10)	0.3483 (3)	0.7321 (3)	0.0207 (8)	
Cl3	0.6854 (3)	0.37701 (8)	0.61594 (7)	0.0291 (3)	
C4	0.4493 (11)	0.2585 (3)	0.7523 (3)	0.0223 (8)	
H4	0.435302	0.211395	0.703291	0.027*	
C5	0.3592 (10)	0.2398 (3)	0.8477 (3)	0.0206 (8)	
Cl5	0.2163 (3)	0.12924 (7)	0.87993 (7)	0.0275 (2)	
C6	0.3861 (10)	0.3095 (3)	0.9187 (3)	0.0209 (8)	
H6	0.332306	0.294833	0.984100	0.025*	

### catena-Poly[[bis(3,5-dichloropyridine)copper(II)]-di-µ-bromido] (1) Atomic displacement parameters (Å^2^)

**Table:**

	*U*^11^	*U*^22^	*U*^33^	*U*^12^	*U*^13^	*U*^23^
Cu1	0.0232 (4)	0.0167 (4)	0.0179 (4)	0.0033 (3)	−0.0022 (3)	−0.0031 (3)
Br1	0.0173 (3)	0.0187 (3)	0.0194 (3)	0.00076 (13)	0.00064 (16)	0.00106 (13)
N1	0.0199 (17)	0.0185 (16)	0.0220 (17)	0.0025 (12)	−0.0007 (13)	−0.0013 (12)
C2	0.0199 (19)	0.0204 (19)	0.0197 (19)	−0.0002 (14)	−0.0007 (14)	−0.0011 (14)
C3	0.0199 (19)	0.0241 (19)	0.0184 (18)	−0.0007 (15)	0.0032 (14)	−0.0022 (14)
Cl3	0.0386 (6)	0.0286 (5)	0.0204 (5)	−0.0038 (4)	0.0073 (4)	−0.0028 (4)
C4	0.026 (2)	0.0204 (19)	0.0207 (19)	0.0015 (16)	−0.0001 (15)	−0.0038 (15)
C5	0.0196 (18)	0.0190 (18)	0.023 (2)	0.0016 (15)	−0.0009 (14)	−0.0013 (15)
Cl5	0.0346 (5)	0.0199 (5)	0.0281 (5)	−0.0047 (4)	0.0021 (4)	−0.0015 (4)
C6	0.0198 (18)	0.0193 (19)	0.0237 (19)	0.0030 (15)	0.0004 (15)	−0.0009 (15)

### catena-Poly[[bis(3,5-dichloropyridine)copper(II)]-di-µ-bromido] (1) Geometric parameters (Å, º)

**Table:**

Cu1—N1^i^	2.039 (3)	C3—C4	1.373 (6)
Cu1—N1	2.039 (3)	C3—Cl3	1.729 (4)
Cu1—Br1	2.4246 (4)	C4—C5	1.390 (6)
Cu1—Br1^i^	2.4246 (4)	C4—H4	0.9500
N1—C2	1.344 (5)	C5—C6	1.391 (6)
N1—C6	1.344 (5)	C5—Cl5	1.725 (4)
C2—C3	1.394 (6)	C6—H6	0.9500
C2—H2	0.9500		
			
N1^i^—Cu1—N1	180.0	C4—C3—C2	120.9 (4)
N1^i^—Cu1—Br1	90.34 (10)	C4—C3—Cl3	120.1 (3)
N1—Cu1—Br1	89.66 (10)	C2—C3—Cl3	119.0 (3)
N1^i^—Cu1—Br1^i^	89.66 (10)	C3—C4—C5	117.0 (4)
N1—Cu1—Br1^i^	90.34 (10)	C3—C4—H4	121.5
Br1—Cu1—Br1^i^	179.999 (15)	C5—C4—H4	121.5
C2—N1—C6	119.5 (3)	C4—C5—C6	120.6 (4)
C2—N1—Cu1	120.2 (3)	C4—C5—Cl5	120.3 (3)
C6—N1—Cu1	120.2 (3)	C6—C5—Cl5	119.1 (3)
N1—C2—C3	120.9 (4)	N1—C6—C5	121.0 (4)
N1—C2—H2	119.5	N1—C6—H6	119.5
C3—C2—H2	119.5	C5—C6—H6	119.5
			
C6—N1—C2—C3	−0.3 (6)	C3—C4—C5—C6	−0.9 (6)
Cu1—N1—C2—C3	−178.9 (3)	C3—C4—C5—Cl5	179.8 (3)
N1—C2—C3—C4	1.9 (6)	C2—N1—C6—C5	−1.9 (6)
N1—C2—C3—Cl3	−178.0 (3)	Cu1—N1—C6—C5	176.7 (3)
C2—C3—C4—C5	−1.3 (6)	C4—C5—C6—N1	2.5 (6)
Cl3—C3—C4—C5	178.7 (3)	Cl5—C5—C6—N1	−178.2 (3)

Symmetry code: (i) −*x*+1, −*y*+1, −*z*+2.

### catena-Poly[[bis(3,5-dichloropyridine)copper(II)]-di-µ-bromido] (1) Hydrogen-bond geometry (Å, º)

**Table:**

*D*—H···*A*	*D*—H	H···*A*	*D*···*A*	*D*—H···*A*
C2—H2···Br1^ii^	0.95	2.83	3.472 (4)	126
C4—H4···Br1^iii^	0.95	2.86	3.622 (4)	138
C6—H6···Br1^iv^	0.95	2.82	3.441 (4)	124

Symmetry codes: (ii) *x*+1, *y*, *z*; (iii) −*x*+1, *y*−1/2, −*z*+3/2; (iv) −*x*, −*y*+1, −*z*+2.

### catena-Poly[[bis(3,5-dimethylpyridine)copper(II)]-di-µ-bromido] (2) Crystal data

**Table:**

[CuBr_2_(C_7_H_9_N)_2_]	*F*(000) = 430
*M**_r_* = 437.66	*D*_x_ = 1.846 Mg m^−^^3^
Monoclinic, *P*2_1_/*c*	Mo *K*α radiation, λ = 0.71073 Å
*a* = 3.9901 (2) Å	Cell parameters from 3415 reflections
*b* = 14.2902 (9) Å	θ = 2.9–28.2°
*c* = 13.8338 (8) Å	µ = 6.45 mm^−^^1^
β = 93.638 (2)°	*T* = 100 K
*V* = 787.20 (8) Å^3^	Needle, green
*Z* = 2	0.46 × 0.03 × 0.03 mm

### catena-Poly[[bis(3,5-dimethylpyridine)copper(II)]-di-µ-bromido] (2) Data collection

**Table:**

Bruker APEXII CCD diffractometer	3425 reflections with *I* > 2σ(*I*)
φ and ω scans	*R*_int_ = 0.058
Absorption correction: multi-scan (*TWINABS*; Sheldrick, 2012)	θ_max_ = 28.3°, θ_min_ = 2.9°
*T*_min_ = 0.650, *T*_max_ = 0.746	*h* = −5→5
3775 measured reflections	*k* = 0→19
3775 independent reflections	*l* = 0→18

### catena-Poly[[bis(3,5-dimethylpyridine)copper(II)]-di-µ-bromido] (2) Refinement

**Table:**

Refinement on *F*^2^	Primary atom site location: structure-invariant direct methods
Least-squares matrix: full	Secondary atom site location: difference Fourier map
*R*[*F*^2^ > 2σ(*F*^2^)] = 0.044	Hydrogen site location: inferred from neighbouring sites
*wR*(*F*^2^) = 0.087	H-atom parameters constrained
*S* = 1.05	*w* = 1/[σ^2^(*F*_o_^2^) + (0.0241*P*)^2^ + 1.7185*P*] where *P* = (*F*_o_^2^ + 2*F*_c_^2^)/3
3775 reflections	(Δ/σ)_max_ = 0.001
91 parameters	Δρ_max_ = 0.69 e Å^−^^3^
0 restraints	Δρ_min_ = −0.64 e Å^−^^3^

### catena-Poly[[bis(3,5-dimethylpyridine)copper(II)]-di-µ-bromido] (2) Special details

**Table:**

Geometry. All esds (except the esd in the dihedral angle between two l.s. planes) are estimated using the full covariance matrix. The cell esds are taken into account individually in the estimation of esds in distances, angles and torsion angles; correlations between esds in cell parameters are only used when they are defined by crystal symmetry. An approximate (isotropic) treatment of cell esds is used for estimating esds involving l.s. planes.
Refinement. Refined as a 4-component twin.Rint = 0.0762 for all 26103 observations and Rint = 0.0577 for all 8044 observations with I > 3sigma(I). Rint is based on agreement between observed single and composite intensities and those calculated from refined unique intensities and twin fractions. 2022 corrected reflections were written to file a HKLF 4-type file. Reflections were merged according to point-group 2/m. Minimum and maximum apparent transmission: 0.649678 0.745686. Additional spherical absorption correction were applied with µ*r = 0.2000. The HKLF 5 dataset was constructed from all observations involving domain 1. 10695 corrected reflections written to HKLF 5-type file. Reflections were merged according to point-group 2/m. Single reflections that also occurred in composites were omitted. Minimum and maximum apparent transmission: 0.649545 0.745686. Additional spherical absorption correction applied with µ*r = 0.2000

### catena-Poly[[bis(3,5-dimethylpyridine)copper(II)]-di-µ-bromido] (2) Fractional atomic coordinates and isotropic or equivalent isotropic displacement parameters (Å^2^)

**Table:**

	*x*	*y*	*z*	*U*_iso_*/*U*_eq_	
Cu1	0.500000	0.500000	0.500000	0.0159 (2)	
Br1	0.88458 (12)	0.40484 (3)	0.60226 (4)	0.01099 (12)	
N1	0.4753 (11)	0.3991 (3)	0.3989 (3)	0.0148 (8)	
C2	0.5684 (13)	0.4157 (4)	0.3100 (4)	0.0151 (11)	
H2	0.644319	0.476702	0.295266	0.018*	
C3	0.5605 (13)	0.3482 (4)	0.2376 (4)	0.0134 (11)	
C4	0.4344 (13)	0.2605 (4)	0.2590 (4)	0.0144 (10)	
H4	0.419115	0.213034	0.210788	0.017*	
C5	0.3306 (13)	0.2424 (4)	0.3513 (4)	0.0147 (11)	
C6	0.3613 (13)	0.3133 (4)	0.4192 (4)	0.0148 (10)	
H6	0.298962	0.300820	0.483049	0.018*	
C7	0.6748 (16)	0.3708 (4)	0.1379 (4)	0.0210 (12)	
H7A	0.651403	0.315085	0.096682	0.025*	
H7B	0.536313	0.421464	0.109048	0.025*	
H7C	0.910514	0.390453	0.143358	0.025*	
C8	0.1978 (15)	0.1474 (4)	0.3771 (4)	0.0205 (12)	
H8A	0.194168	0.106560	0.320159	0.025*	
H8B	0.343355	0.120063	0.429397	0.025*	
H8C	−0.030447	0.153981	0.398525	0.025*	

### catena-Poly[[bis(3,5-dimethylpyridine)copper(II)]-di-µ-bromido] (2) Atomic displacement parameters (Å^2^)

**Table:**

	*U*^11^	*U*^22^	*U*^33^	*U*^12^	*U*^13^	*U*^23^
Cu1	0.0231 (4)	0.0114 (4)	0.0122 (4)	0.0079 (4)	−0.0074 (4)	−0.0055 (3)
Br1	0.01092 (18)	0.0107 (2)	0.0112 (2)	0.0017 (2)	−0.00078 (16)	0.0002 (2)
N1	0.0191 (19)	0.012 (2)	0.013 (2)	0.0028 (18)	−0.0045 (18)	−0.0028 (16)
C2	0.016 (2)	0.012 (2)	0.016 (2)	0.002 (2)	−0.003 (2)	−0.003 (2)
C3	0.014 (3)	0.015 (2)	0.011 (2)	0.003 (2)	0.001 (2)	−0.002 (2)
C4	0.012 (2)	0.014 (2)	0.017 (3)	0.002 (2)	−0.004 (2)	−0.0072 (19)
C5	0.013 (3)	0.014 (2)	0.017 (3)	0.002 (2)	0.000 (2)	−0.004 (2)
C6	0.014 (2)	0.016 (2)	0.014 (2)	0.001 (2)	0.000 (2)	0.001 (2)
C7	0.023 (3)	0.022 (3)	0.019 (3)	−0.001 (2)	0.005 (2)	−0.001 (2)
C8	0.020 (3)	0.020 (3)	0.022 (3)	−0.005 (2)	0.001 (2)	−0.001 (2)

### catena-Poly[[bis(3,5-dimethylpyridine)copper(II)]-di-µ-bromido] (2) Geometric parameters (Å, º)

**Table:**

Cu1—N1	2.007 (4)	C4—H4	0.9500
Cu1—N1^i^	2.007 (4)	C5—C6	1.382 (7)
Cu1—Br1	2.4350 (5)	C5—C8	1.507 (7)
Cu1—Br1^i^	2.4350 (5)	C6—H6	0.9500
N1—C2	1.328 (7)	C7—H7A	0.9800
N1—C6	1.343 (7)	C7—H7B	0.9800
C2—C3	1.389 (7)	C7—H7C	0.9800
C2—H2	0.9500	C8—H8A	0.9800
C3—C4	1.390 (7)	C8—H8B	0.9800
C3—C7	1.515 (7)	C8—H8C	0.9800
C4—C5	1.391 (8)		
			
N1—Cu1—N1^i^	180.0 (2)	C6—C5—C4	117.9 (5)
N1—Cu1—Br1	90.27 (12)	C6—C5—C8	121.1 (5)
N1^i^—Cu1—Br1	89.73 (12)	C4—C5—C8	121.0 (5)
N1—Cu1—Br1^i^	89.73 (12)	N1—C6—C5	123.0 (5)
N1^i^—Cu1—Br1^i^	90.27 (12)	N1—C6—H6	118.5
Br1—Cu1—Br1^i^	180.0	C5—C6—H6	118.5
C2—N1—C6	118.3 (4)	C3—C7—H7A	109.5
C2—N1—Cu1	120.9 (4)	C3—C7—H7B	109.5
C6—N1—Cu1	120.9 (3)	H7A—C7—H7B	109.5
N1—C2—C3	123.3 (5)	C3—C7—H7C	109.5
N1—C2—H2	118.3	H7A—C7—H7C	109.5
C3—C2—H2	118.3	H7B—C7—H7C	109.5
C2—C3—C4	117.6 (5)	C5—C8—H8A	109.5
C2—C3—C7	121.0 (5)	C5—C8—H8B	109.5
C4—C3—C7	121.3 (5)	H8A—C8—H8B	109.5
C3—C4—C5	119.8 (4)	C5—C8—H8C	109.5
C3—C4—H4	120.1	H8A—C8—H8C	109.5
C5—C4—H4	120.1	H8B—C8—H8C	109.5
			
C6—N1—C2—C3	−1.4 (8)	C3—C4—C5—C6	−0.7 (8)
Cu1—N1—C2—C3	179.2 (4)	C3—C4—C5—C8	−179.1 (5)
N1—C2—C3—C4	2.9 (8)	C2—N1—C6—C5	−1.3 (8)
N1—C2—C3—C7	−179.1 (5)	Cu1—N1—C6—C5	178.1 (4)
C2—C3—C4—C5	−1.7 (8)	C4—C5—C6—N1	2.3 (8)
C7—C3—C4—C5	−179.8 (5)	C8—C5—C6—N1	−179.3 (5)

Symmetry code: (i) −*x*+1, −*y*+1, −*z*+1.

### catena-Poly[[bis(3,5-dimethylpyridine)copper(II)]-di-µ-bromido] (2) Hydrogen-bond geometry (Å, º)

**Table:**

*D*—H···*A*	*D*—H	H···*A*	*D*···*A*	*D*—H···*A*
C2—H2···Br1^i^	0.95	3.12	3.406 (5)	100
C6—H6···Br1^ii^	0.95	2.83	3.515 (5)	130

Symmetry codes: (i) −*x*+1, −*y*+1, −*z*+1; (ii) *x*−1, *y*, *z*.
